# The combination of soluble forms of PD-1 and PD-L1 as a predictive marker of PD-1 blockade in patients with advanced cancers: a multicenter retrospective study

**DOI:** 10.3389/fimmu.2023.1325462

**Published:** 2023-12-11

**Authors:** Takashi Kurosaki, Kenji Chamoto, Shinichiro Suzuki, Hiroaki Kanemura, Seiichiro Mitani, Kaoru Tanaka, Hisato Kawakami, Yo Kishimoto, Yasuharu Haku, Katsuhiro Ito, Toshiyuki Sato, Chihiro Suminaka, Mami Yamaki, Yasutaka Chiba, Tomonori Yaguchi, Koichi Omori, Takashi Kobayashi, Kazuhiko Nakagawa, Tasuku Honjo, Hidetoshi Hayashi

**Affiliations:** ^1^ Department of Medical Oncology, Kindai University Faculty of Medicine, Osaka-Sayama, Japan; ^2^ Department of Immunology and Genomic Medicine, Center for Cancer Immunotherapy and Immunobiology, Kyoto University Graduate School of Medicine, Kyoto, Japan; ^3^ Department of Immuno-Oncology PDT, Kyoto University Graduate School of Medicine, Kyoto, Japan; ^4^ Department of Otolaryngology–Head and Neck Surgery, Graduate School of Medicine, Kyoto University, Kyoto, Japan; ^5^ Department of Urology, Kyoto University Graduate School of Medicine, Kyoto, Japan; ^6^ Central Research Laboratories, Sysmex Corporation, Kobe, Japan; ^7^ Business Strategy Development, Sysmex Corporation, Kobe, Japan; ^8^ Clinical Research Center, Kindai University Hospital, Osaka-Sayama, Japan

**Keywords:** immune checkpoint inhibitor, nivolumab, pembrolizumab, soluble PD-1, soluble PD-L1

## Abstract

**Introduction:**

The clinical relevance of soluble forms of programmed cell death-1 (sPD-1) and programmed cell death-ligand 1 (sPD-L1) remains unclear. We here investigated the relation between the efficacy of PD-1 blockade and pretreatment plasma levels of sPD-1 and sPD-L1 across a broad range of cancer types.

**Methods:**

We retrospectively analyzed clinical data from 171 patients with advanced solid tumors who received nivolumab or pembrolizumab monotherapy regardless of treatment line. The concentrations of sPD-1 and sPD-L1 were measured with a fully automated immunoassay (HISCL system).

**Results:**

The study subjects comprised patients with head and neck cancer (*n* = 50), urothelial cancer (*n* = 42), renal cell cancer (*n* = 37), gastric cancer (*n* = 20), esophageal cancer (*n* = 10), malignant pleural mesothelioma (*n* = 6), or microsatellite instability-high tumors (*n* = 6). High or low levels of sPD-1 or sPD-L1 were not significantly associated with progression-free survival (PFS) or overall survival (OS) for PD-1 blockade in the entire study population. Comparison of treatment outcomes according to combinations of high or low sPD-1 and sPD-L1 levels, however, revealed that patients with low sPD-1 and high sPD-L1 concentrations had a significantly poorer PFS (HR of 1.79 [95% CI, 1.13–2.83], *p* = 0.01) and a tendency toward poorer OS (HR of 1.70 [95% CI, 0.99–2.91], *p* = 0.05) compared with all other patients.

**Conclusion:**

Our findings suggest that the combination of low sPD-1 and high sPD-L1 levels is a potential negative biomarker for PD-1 blockade therapy.

## 1 Introduction

Despite the substantial improvements in cancer treatment in recent decades, advanced solid tumors diagnosed at unresectable or recurrent stages still have a poor prognosis and remain the leading cause of death worldwide (1). The development of new systemic therapies that are effective across cancer types is therefore a pressing need.

Immune checkpoint inhibitors (ICIs) are new therapeutic agents that target co-inhibitory molecules expressed on T lymphocytes and which enhance antitumor immunity (2). In particular, antibodies to programmed cell death–1 (PD-1) that block the function of this negative regulatory molecule on T cells are the most widely administered type of ICI and have revolutionized the treatment of advanced malignancies (3). However, the survival outcome for treatment with PD-1 antibodies remains unsatisfactory overall, and the greatest benefit of such treatment is restricted to just a few cancer types. Tumor-agnostic biomarkers that predict the efficacy of PD-1 blockade therapy are therefore needed for optimal patient selection.

One promising such biomarker, programmed cell death–ligand 1 (PD-L1) expression on tumor or immune cells, has been widely investigated. Whereas an association between PD-L1 expression and clinical response has been detected for specific tumor types such as non–small cell lung cancer (NSCLC), results from several prospective trials suggest that PD-L1 expression may not be a robust predictor of the response to PD-1 antibodies in all cancer types (3–6). Possible explanations for this lack of robustness include intratumoral heterogeneity and the dynamic nature of the tumor microenvironment (TME) (7). Compared with biopsy specimens that represent just a fraction of the entire TME, peripheral blood samples are thought to reflect more of the TME and therefore might be a better option for biomarker detection. Blood testing also has the advantages of being minimally invasive and providing dynamic assessments in real time.

In addition to their expression at the cell surface, the receptors and ligands that function as immune checkpoint molecules are present as soluble forms in the circulation (8, 9). Regarding PD-L1, it was reported that the correlation between the serum levels of soluble form and the tumor PD-L1 expression was weak in patients with NSCLC (10), thus soluble forms of immune checkpoint molecules have potential to be a biomarker independent of those of membranous expression. The levels of such soluble forms of PD-1 (sPD-1) and PD-L1 (sPD-L1) have been found to be related to the progression and prognosis of PD-1 blockade therapy, but only a limited number of such studies has focused on advanced solid tumors other than NSCLC and melanoma (11). The aim of the present study was to investigate the possible relation between the efficacy of PD-1 blockade therapy and pretreatment plasma levels of sPD-1 and sPD-L1 across a broad range of advanced cancers that had limited clinical focus.

## 2 Materials and methods

### 2.1 Patients

Patients were enrolled in this study if (1) they had a solid tumor at an advanced stage other than NSCLC or melanoma and were not eligible for curative treatment, (2) they had been treated with PD-1 antibody monotherapy regardless of treatment line, and (3) a blood sample collected before the start of PD-1 blockade therapy and clinicopathologic data were available. Patients were retrospectively identified from those attending Kindai University Hospital or Kyoto University Hospital. The study was conducted according to the Declaration of Helsinki, and the protocols were approved by the Institutional Review Board of each participating hospital.

### 2.2 Data collection

Clinicopathologic data—including sex, age, ECOG performance status, histological subtype, and white blood cell differential for a peripheral blood smear collected at the time of the first PD-1 antibody administration—were obtained from medical records. The neutrophil/lymphocyte ratio (NLR), which has been implicated as a predictive biomarker of ICI treatment outcome (12–14), was calculated for before PD-1 blockade therapy, with a value of 5 being specified as the cutoff between a high and low NLR as in previous studies (12–14). Treatment history and the therapeutic effect of the PD-1 antibody were also retrieved. Tumor response was assessed according to RECIST version 1.1 (15). Overall response rate was defined as the proportion of patients with a complete or partial response as the best overall response, which was assessed regardless of the presence of measurable disease. Progression-free survival (PFS) was measured from the time of treatment initiation to clinical or radiographic progression or death from any cause. Overall survival (OS) was measured from the time of treatment initiation to death from any cause. Patients without documented clinical or radiographic disease progression or who were still alive were censored at the last follow-up.

### 2.3 Measurement of sPD-1 and sPD-L1 levels

ELISAs have been adopted for the measurement of sPD-1 and sPD-L1 concentrations in many previous studies but have limited precision and reproducibility because of the manual procedures involved (16, 17). To overcome these limitations, we used a fully automated immunoassay system based on chemiluminescent magnetic technology (HISCL system), which is rapid, sensitive, and reproducible and is able to measure sPD-1 and sPD-L1 levels accurately (11, 17). Plasma samples obtained before PD-1 antibody treatment were considered appropriate for this study; if plasma samples were not available, serum samples were permitted. The high concordance between plasma and serum concentrations was confirmed by Sysmex Corporation, the device provider and a study collaborator, with the use of commercially available paired samples (**Figure S1**).

### 2.4 Statistical analysis

Cutoff values for sPD-1 and sPD-L1 concentrations were defined as the median for each cancer type, so that survival analysis according to the soluble markers would not be affected by the potential difference in distributions of sPD-1 and sPD-L1 concentrations among cancer types. The outcome of PD-1 blockade therapy was compared between patients with high or low circulating levels of sPD-1 or sPD-L1. Pairwise comparisons of sPD-1 and sPD-L1 levels were also performed. PFS and OS curves were constructed by the Kaplan-Meier method. Between-group differences in survival analyses were assessed with the log-rank test. The hazard ratio (HR) and its 95% confidence interval (CI) were determined with the use of a Cox proportional hazard regression model. Adjustment for possible confounding factors was performed with a multivariable regression model including explanatory variables with a *p* value of <0.1 in univariable analysis. A two-sided *p* value of <0.05 was considered statistically significant. All statistical analysis was performed with Stata BE version 17.0 (StataCorp) or GraphPad Prism 9.0 software.

## 3 Results

### 3.1 Characteristics of the study population

A total of 171 patients with solid tumors were enrolled in the study, with their clinical and pathological features being summarized in **Table 1**. The most common cancer type was head and neck cancer (*n* = 50, 29.2%), followed by urothelial cancer (*n* = 42, 24.6%), renal cell cancer (*n* = 37, 21.6%), gastric cancer (*n* = 20, 11.7%), esophageal cancer (*n* = 10, 5.8%), malignant pleural mesothelioma (*n* = 6, 3.5%), and microsatellite instability (MSI)–high solid tumors (*n* = 6, 3.5%). The major histological subtypes included squamous cell carcinoma (*n* = 54, 31.6%), urothelial carcinoma (*n* = 42, 24.6%), clear cell carcinoma (*n* = 37, 21.6%), adenocarcinoma (*n* = 26, 15.2%), and others (*n* = 12, 7.0%). Of the 171 patients, 121 (70.8%) individuals were treated with nivolumab monotherapy and 50 (29.2%) with pembrolizumab monotherapy. Almost all patients (*n* = 169, 98.8%) received systemic therapy before PD-1 blockade therapy.

**Table 1:** Patient characteristics.

Characteristic	No. of patients	%
[Median age (range), years	70 (27–89)]	
Sex Male Female	123 48	71.9 28.1
Cancer type Head and neck cancer Urothelial cancer Renal cell cancer Gastric cancer Esophageal cancer Malignant pleural mesothelioma MSI-high solid tumors^a^	50 42 37 20 10 6 6	29.2 24.6 21.6 11.7 5.8 3.5 3.5
Histological subtype Squamous cell carcinoma Urothelial carcinoma Clear cell carcinoma Adenocarcinoma Epithelioid mesothelioma Sarcomatoid mesothelioma Adenoid cystic carcinoma Neuroendocrine carcinoma Salivary duct carcinoma Not otherwise specified	54 42 37 26 3 3 1 1 1 3	31.6 24.6 21.6 15.2 1.8 1.8 0.6 0.6 0.6 1.8
ECOG performance status 0 1 2 3 4	46 100 17 7 1	26.9 58.5 9.9 4.1 0.6
Number of prior systemic therapies 0 1 ≥2	2 112 57	1.2 65.5 33.3
ICI regimen Nivolumab Pembrolizumab	121 50	70.8 29.2

MSI, microsatellite instability; ICI, immune checkpoint inhibitor.

a MSI-high solid tumors included colorectal cancer (n = 4), cancer of unknown primary (n = 1), and bile duct cancer (n = 1).

### 3.2 Relation between soluble markers and baseline characteristics

**Figure 1:**
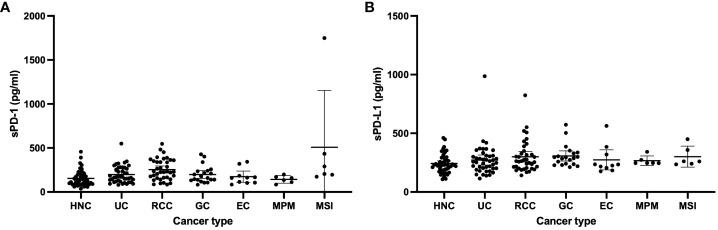
Levels of sPD-1 **(A)** and sPD-L1 **(B)** in patients with head and neck cancer (HNC, *n* = 50), urothelial cancer (UC, *n* = 42), renal cell cancer (RCC, *n* = 37) gastric cancer (GC, *n* = 20), esophageal cancer (EC, *n* = 10), malignant pleural mesothelioma (MPM, *n* = 6), or microsatellite instability (MSI)–high cancer (*n* = 6).

For the total patient population, the median circulating sPD-1 and sPD-L1 concentrations were 169 pg/ml (interquartile range [IQR], 112–257) and 248 pg/ml (IQR, 211–310), respectively. The distribution of sPD-1 and sPD-L1 levels for each cancer type is shown in **Figure 1**. The median sPD-1 and sPD-L1 concentrations were 132 pg/ml (IQR, 87–201) and 233 pg/ml (IQR, 192–283) for head and neck cancer, 170 pg/ml (IQR, 121–276) and 256 pg/ml (IQR, 200–309) for urothelial cancer, 229 pg/ml (IQR, 163–351) and 263 pg/ml (IQR, 205–326) for renal cell cancer, 170 pg/ml (IQR, 135–238) and 291 pg/ml (IQR, 239–333) for gastric cancer, 161 pg/ml (IQR, 106–183) and 230 pg/ml (IQR, 206–319) for esophageal cancer, 148 pg/ml (IQR, 112–171) and 247 pg/ml (IQR, 246–273) for malignant pleural mesothelioma, and 247 pg/ml (IQR, 196–434) and 261 pg/ml (IQR, 241–358) for MSI-high solid tumors, respectively. The relation between clinical features of the patients and sPD-1 and sPD-L1 levels is summarized in **Table 2**. The median sPD-1 level was significantly higher in elderly patients, whereas the median sPD-L1 level was significantly higher in patients who were elderly and male and had a poor ECOG performance status (≥2).

**Table 2:** Circulating sPD-1 and sPD-L1 levels of study patients according to baseline characteristics.

Characteristic	sPD-1		sPD-L1	
	Median [IQR], pg/ml	*p*	Median [IQR], pg/ml	*p*
Age (years) ≥70 <70	185 [142–276] 148 [93–228]	<0.01	260 [230–318] 234 [184–298]	0.02
Sex Female Male	166 [119–264] 169 [112–257]	0.78	235 [190–279] 258 [218–319]	0.02
ECOG performance status ≥2 0 or 1	203 [148–284] 163 [111–246]	0.11	302 [261–369] 241 [204–300]	0.02
No. of previous regimens ≥2 0 or 1	188 [136–320] 164 [109–235]	0.07	256 [220–329] 247 [204–302]	0.33

sPD-1, soluble programmed cell death–1; sPD-L1, soluble programmed cell death–ligand 1; IQR, interquartile range.

The p values were determined with the Wilcoxon ranked sum test.

### 3.3 Relation between soluble markers and treatment efficacy

**Figure 2:**
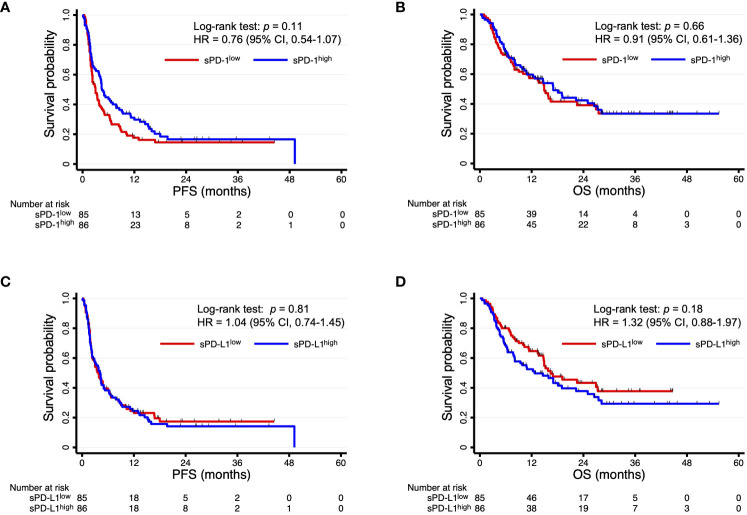
Kaplan-Meier curves of PFS **(A, C)** and OS **(B, D)** for patients with sPD-1^high^ or sPD-1^low^ levels **(A, B)** or with sPD-L1^high^ or sPD-L1^low^ levels **(C, D)**.

Among the 171 patients, there were 96 deaths and 138 disease progression events after the onset of PD-1 blockade therapy with a median follow-up time of 11.4 months. Kaplan-Meier curves for PFS and OS were constructed according to circulating sPD-1 and sPD-L1 levels in order to evaluate their independent predictive values (**Figure 2**). Patients with high sPD-1 (sPD-1^high^) levels had a numerically longer PFS relative to those with low sPD-1 (sPD-1^low^) levels (median of 4.5 vs. 3.0 months; HR of 0.76, with a 95% CI of 0.54–1.07; *p* = 0.11), although the difference was not statistically significant (**Figure 2A**). The circulating concentration of sPD-1 was also not significantly associated with OS (**Figure 2B**). In addition, no significant association was apparent between sPD-L1 levels and either PFS (**Figure 2C**) or OS (**Figure 2D**), although OS tended to be shorter in patients with sPD-L1^high^ concentrations relative to those with sPD-L1^low^ concentrations (median of 12.7 vs. 16.4 months, HR of 1.32 [95% CI, 0.88–1.97], *p* = 0.18). Subgroup analysis according to each of the three most common cancer types was shown in **Figures S2**, **S3**.

**Figure 3:**
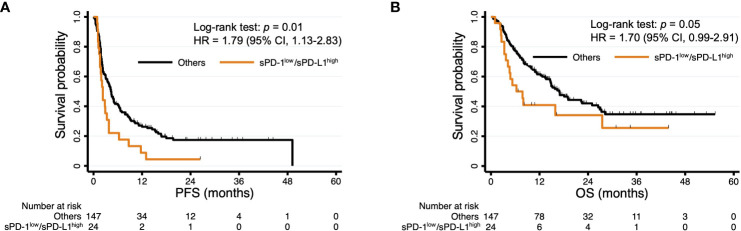
Kaplan-Meier curves of PFS **(A)** and OS **(B)** for patients with sPD-1^low^/sPD-L1^high^ levels and all other patients. The curves for sPD-1^low^/sPD-L1^high^ are the same as those in **Figure S2**.

We next hypothesized that the accuracy of survival prediction might be increased by combining sPD-1 and sPD-L1 levels. Indeed, we found that patients with both sPD-1^low^ and sPD-L1^high^ concentrations tended to have a shorter PFS and OS compared with each of the other three groups of patients based on paired sPD-1 and sPD-L1 levels (**Figure S4**). The patients with sPD-1^low^/sPD-L1^high^ levels had a significantly shorter PFS (median of 2.3 vs. 4.3 months, HR of 1.79 [95% CI, 1.13–2.83], *p* = 0.01) and a numerically shorter OS (median of 6.3 vs. 16.9 months, HR of 1.70 [95% CI, 0.99–2.91], *p* = 0.05) compared with the other groups of patients combined (**Figure 3**). We then performed multivariable analysis to eliminate bias from possible confounding factors. We adopted NLR and cancer type (renal cell cancer or not, and gastric cancer or not) as explanatory variables for PFS, and sex, ECOG performance status, NLR, and cancer type (urothelial cancer or not, renal cell cancer or not, gastric cancer or not, and MSI-high cancer or not) as those for OS, on the basis of univariable analysis (**Table 3**). The sPD-1^low^/sPD-L1^high^ combination was significantly associated with not only PFS (HR of 1.62 [95% CI, 1.03–2.58], *p* = 0.04) but also OS (HR of 1.86 [95% CI, 1.06–3.26], *p* = 0.03) (**Table 4**). Patients with sPD-1^low^/sPD-L1^high^ levels also had a numerically lower overall response rate compared with the other patients (16.7% vs. 34.7%, *p* = 0.08 [Chi-squared test]).

**Table 3:** Univariable analysis of progression-free survival (PFS) and overall survival (OS).

Characteristic	PFS			OS		
	HR	95% CI	*p*	HR	95% CI	*p*
Age ≥70 years (vs. <70)	1.04	0.74–1.45	0.84	0.96	0.64–1.44	0.85
Sex Female (vs. male)	0.93	0.63–1.36	0.70	0.63	0.38–1.03	0.06
Cancer type (vs. others) Head and neck cancer Urothelial cancer Renal cell cancer Gastric cancer Esophageal cancer Malignant pleural mesothelioma MSI-high solid tumors	0.94 1.02 0.6 3.7 1.47 1.68 0.4	0.65–1.38 0.69–1.50 0.39–0.91 2.23–6.12 0.77–2.81 0.74–3.84 0.13–1.27	0.76 0.93 0.01 <0.01 0.23 0.21 0.12	1.05 1.93 0.27 3.01 0.95 1.13 0.21	0.68–1.62 1.25–2.98 0.14–0.50 1.76–5.15 0.39–2.35 0.36–3.58 0.03–1.51	0.83 <0.01 <0.01 <0.01 0.92 0.83 0.09
ECOG performance status ≥2 (vs. 0 or 1)	1.32	0.83–2.11	0.23	2.33	1.39–3.90	<0.01
No. of previous regimens ≥2 (vs. 0 or 1)	1.22	0.86–1.74	0.26	0.99	0.65–1.52	0.97
Neutrophil/lymphocyte ratio ≥5 (vs. <5)	1.45	1.09–1.93	0.03	1.88	1.40–2.52	<0.01
Soluble markers sPD-1^low^/sPD-L1^high^ (vs. others)	1.79	1.13–2.83	0.01	1.70	0.99–2.91	0.05

HR, hazard ratio; CI, confidence interval; MSI, microsatellite instability; sPD-1, soluble programmed cell death–1; sPD-L1, soluble programmed cell death–ligand 1.

**Table 4:** Multivariable analysis of progression-free survival (PFS) and overall survival (OS).

Characteristic	PFS			OS		
	HR	95% CI	*p*	HR	95% CI	*p*
Sex Female (vs. male)				1.07	0.64–1.80	0.79
Cancer type (vs. others) Urothelial cancer Renal cell cancer Gastric cancer MSI-high solid tumors	0.67 3.63	0.44–1.03 2.14–6.16	0.07 <0.01	1.37 0.28 3.06 0.23	0.80–2.35 0.14–0.57 1.65–5.66 0.03–1.71	0.25 <0.01 <0.01 0.15
ECOG performance status ≥2 (vs. 0 or 1)				2.17	1.22–3.86	<0.01
Neutrophil/lymphocyte ratio ≥5 (vs. <5)	1.59	1.20–2.10	<0.01	1.93	1.42–2.62	<0.01
Soluble markers sPD-1^low^/sPD-L1^high^ (vs. others)	1.62	1.03–2.58	0.04	1.86	1.06–3.26	0.03

HR, hazard ratio; CI, confidence interval; MSI, microsatellite instability; sPD-1, soluble programmed cell death–1; sPD-L1, soluble programmed cell death–ligand 1.

**Figure 4:**
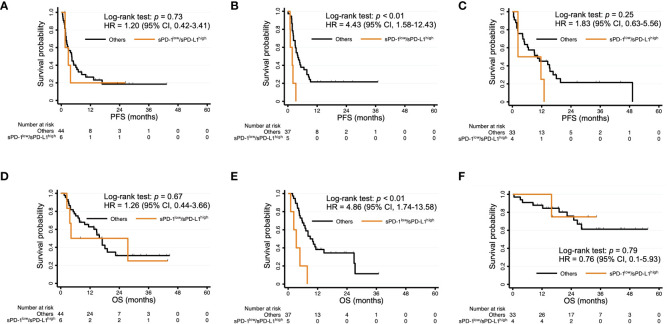
Kaplan-Meier curves of PFS **(A–C)** and OS **(D–F)** for patients with sPD-1^low^/sPD-L1^high^ levels and the other patients among individuals with head and neck cancer **(A, D)**, urothelial cancer **(B, E)**, or renal cell cancer **(C, F)**.

Finally, we conducted subgroup analysis for PFS and OS according to cancer type. The comparisons between sPD-1^low^/sPD-L1^high^ patients and the other patients for each of the three most common cancer types in the study population are shown in **Figure 4**. The sPD-1^low^/sPD-L1^high^ combination was significantly associated with a shorter PFS and OS among patients with urothelial cancer (**Figure 4B, E**), whereas it was not significantly associated with PFS or OS for those with head and neck cancer (**Figure 4A, D**) or renal cell cancer (**Figure 4C, F**).

## 4 Discussion

As far as we are aware, the present study is the first to comprehensively assess pretreatment sPD-1 and sPD-L1 levels across a broad range of advanced cancer types for patients treated with a PD-1 antibody. A notable feature of our study is the use of the HISCL system, a fully automated immunoassay with a high sensitivity and reproducibility, for measurement of the soluble markers (11, 17). We found that the combination of low sPD-1 and high sPD-L1 concentrations was associated with a shorter PFS and OS for patients with advanced solid tumors treated with nivolumab or pembrolizumab monotherapy.

Soluble PD-L1 in the circulation is thought to be produced as a result of alternative mRNA splicing or proteolytic cleavage of PD-L1 at the cell surface in tumor cells or mature dendric cells (18–20). Previous studies have found that high sPD-L1 levels at baseline were associated with a poor PFS and OS in patients treated with ICIs (10, 21–23). One possible explanation for this negative relation between sPD-L1 levels and ICI efficacy is that sPD-L1 binds to PD-1 on the surface of T lymphocytes and thereby disrupts their activation and induces apoptosis (11, 24, 25). It has also been proposed that sPD-L1 might act competitively with PD-1 antibodies and thereby attenuate their pharmacological action (26). In the present study, patients with sPD-L1^high^ levels tended to have a shorter OS, consistent with previous results. However, we considered that sPD-L1 alone was not sufficient for robust prediction of the outcome of PD-1 blockade therapy, given that we did not detect a difference in PFS between sPD-L1^high^ and sPD-L1^low^ patients.

Soluble PD-1 is thought to be generated primarily by alternative splicing of the *PDCD1* gene (27). Although the role of sPD-1 has not been fully elucidated, several preclinical studies have suggested that it promotes the activation of T lymphocytes and enhances the antitumor immune response (11, 28, 29), possibly through suppression of the interaction between PD-1 at the cell surface and its ligands. Clinical studies that have examined the association between sPD-1 levels and survival outcome of immune checkpoint blockade have reported inconsistent findings. A retrospective study of metastatic NSCLC patients treated with nivolumab found that high baseline sPD-1 levels were associated with a shorter PFS in univariable analysis (30). Another study reported that pretreatment sPD-1 levels were not related to either PFS or OS for advanced NSCLC patients treated with ICIs either alone or together with cytotoxic chemotherapy (31). In a study of patients with advanced melanoma, an increase in the sPD-1 concentration after the onset of treatment was a strong individual predictor of a better PFS for nivolumab plus ipilimumab, an antibody to cytotoxic T lymphocyte–associated protein–4 (CTLA-4), implicating sPD-1 in the activation of CD8^+^ T lymphocytes and the antitumor immune response (32). High sPD-1 levels might be a negative predictor for PD-1 blockade therapy if sPD-1 acts as a decoy for PD-1 antibodies and thereby attenuates their action in the TME. However, elevated sPD-1 levels might also be considered a favorable factor for ICI treatment if sPD-1 inhibits the interaction between PD-1 and PD-L1 (11, 28–30). Further preclinical investigation is warranted to determine the influence of sPD-1 in the TME and its interaction with ICIs. We here found that sPD-L1^high^ levels were significantly associated with a poor PFS only in sPD-1^low^ patients, suggesting that a favorable effect of sPD-1^high^ levels on antitumor immunity might attenuate a negative impact of sPD-L1^high^ levels on CD8^+^ T lymphocyte activation. A recent study showed that a low sPD-1/sPD-L1 ratio at baseline was associated with a shorter OS in comparison with a high sPD-1/sPD-L1 ratio in patients with advanced melanoma treated with nivolumab or pembrolizumab (33), consistent with our present results.

We found that the sPD-1^low^/sPD-L1^high^ combination was independently associated with shorter OS as well as shorter PFS after adjustment for confounding factors in our multivariable model. In the uni- and multivariable analyses, we treated cancer types as explanatory variables, given that our study targeted a variety of advanced solid tumors. Our findings thus suggest that the sPD-1^low^/sPD-L1^high^ combination is a promising candidate for a biomarker associated with poor efficacy of PD-1 blockade therapy across cancer types.

There are several limitations to our retrospective study. First, it lacked a validation cohort to confirm the adequacy of the selected cutoff values for sPD-1 and sPD-L1 levels. Second, it lacked a control patient group treated with chemotherapeutic or molecularly targeted agents with different mechanisms of action from ICIs, making it difficult to determine whether our observations are specific to PD-1 antibodies. The study subjects also did not receive ICI treatment other than PD-1 antibody monotherapy. Given that combinations of PD-1 or PD-L1 antibodies with a CTLA-4 antibody, or of chemotherapy with immunotherapy, have recently become important treatment options for several advanced malignancies, additional investigation is warranted to assess the relation between sPD-1^low^/sPD-L1^high^ levels and the efficacy of these combination therapies in the front line. Third, the aim of this study was to explore the overall trends of sPD-1 and sPD-L1 across a broad range of cancer types, and the sample size was insufficient to permit a detailed analysis on the specific cancer types under consideration.

In conclusion, our findings suggest that the combination of low sPD-1 and high sPD-L1 levels at baseline is a potential negative biomarker of PFS and OS for PD-1 antibody monotherapy in a variety of cancer types. Prospective evaluation will be needed to validate and confirm our observations.

## Data availability statement

The raw data supporting the conclusions of this article will be made available by the authors, without undue reservation.

## Ethics statement

The studies involving humans were approved by Human Genome/Gene Analysis Research Ethics Committee of Kindai University Faculty of Medicine. The studies were conducted in accordance with the local legislation and institutional requirements. The participants provided their written informed consent to participate in this study.

## Author contributions

TKu: Writing – original draft, Writing – review &amp; editing, Conceptualization, Data curation, Formal Analysis, Investigation, Methodology, Project administration, Resources, Software. KC: Conceptualization, Data curation, Formal Analysis, Investigation, Methodology, Writing – original draft, Writing – review & editing. SS: Data curation, Resources, Writing – review & editing. HKan: Data curation, Resources, Writing – review & editing. SM: Data curation, Resources, Writing – review & editing. KT: Data curation, Resources, Writing – review & editing. HKaw: Data curation, Resources, Writing – review & editing. YK: Data curation, Resources, Writing – review & editing. YH: Data curation, Resources, Writing – review & editing. KI: Data curation, Resources, Writing – review & editing. TS: Conceptualization, Data curation, Formal Analysis, Funding acquisition, Investigation, Methodology, Project administration, Resources, Writing – original draft, Writing – review & editing. CS: Conceptualization, Data curation, Formal Analysis, Funding acquisition, Investigation, Methodology, Project administration, Resources, Writing – original draft, Writing – review & editing. MY:Conceptualization, Data curation, Formal Analysis, Funding acquisition, Investigation, Methodology, Project administration, Resources, Writing – original draft, Writing – review & editing. YC: Formal Analysis, Investigation, Methodology, Writing – original draft, Writing – review & editing. TY: Data curation, Resources, Writing – review & editing. KO: Data curation, Resources, Writing – review & editing. TKo: Data curation, Resources, Writing – review & editing. KN: Supervision, Writing – review & editing. TH: Supervision, Writing – review & editing. HH: Conceptualization, Formal Analysis, Funding acquisition, Investigation, Methodology, Project administration, Writing – original draft, Writing – review & editing.

## Supplementary material

Supplementary Figure 1 Scatter plot and linear regression for the plasma and serum concentrations of sPD-1 **(A)** or sPD-L1 **(B)** in matched paired samples. Correlation was evaluated with the Spearman correlation test.

Supplementary Figure 2 Kaplan-Meier curves of PFS **(A–C)** and OS **(D–F)** for patients with sPD-1^high^ or sPD-1^low^ levels among individuals with head and neck cancer **(A, D)**, urothelial cancer **(B, E)**, or renal cell cancer **(C, F)**.

Supplementary Figure 3 Kaplan-Meier curves of PFS **(A–C)** and OS **(D–F)** for patients with sPD-L1^high^ or sPD-L1^low^ levels among individuals with head and neck cancer **(A, D)**, urothelial cancer **(B, E)**, or renal cell cancer **(C, F)**.

Supplementary Figure 4 Kaplan-Meier curves of PFS **(A)** and OS **(B)** for all patients according to combined low or high levels of sPD-1 and sPD-L1.
