# Supplementary figures and images for: The combination of soluble forms of PD-1 and PD-L1 as a predictive marker of PD-1 blockade in patients with advanced cancers: a multicenter retrospective study
Source: Front Immunol. 2023 Dec 11;14:1325462. doi: 10.3389/fimmu.2023.1325462 (PMC10750355; doi:10.3389/fimmu.2023.1325462)

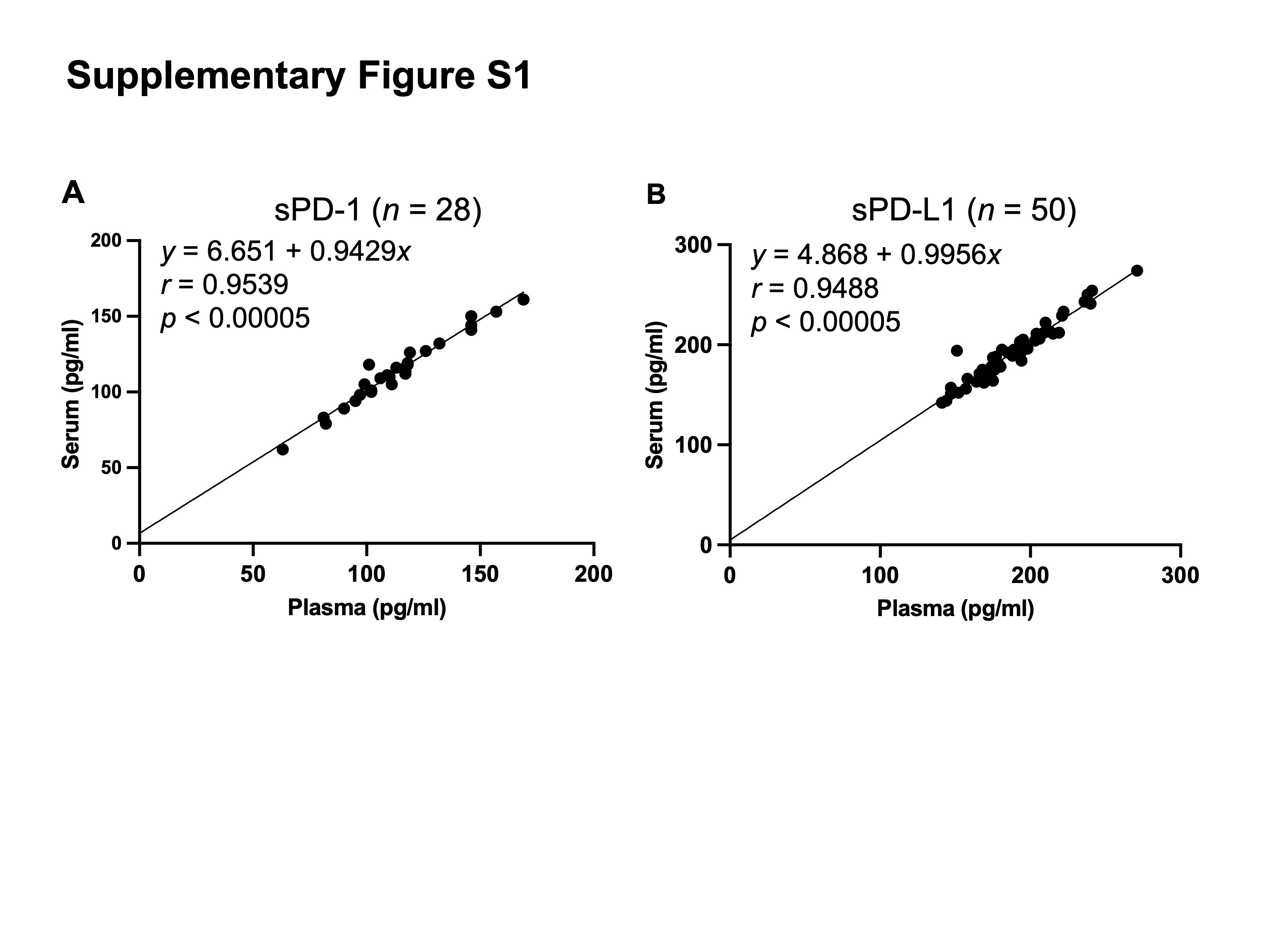

Supplement: Supplementary Figure 1 — Scatter plot and linear regression for the plasma and serum concentrations of sPD-1 (A) or sPD-L1 (B) in matched paired samples. Correlation was evaluated with the Spearman correlation test. [file Image_1.jpeg]

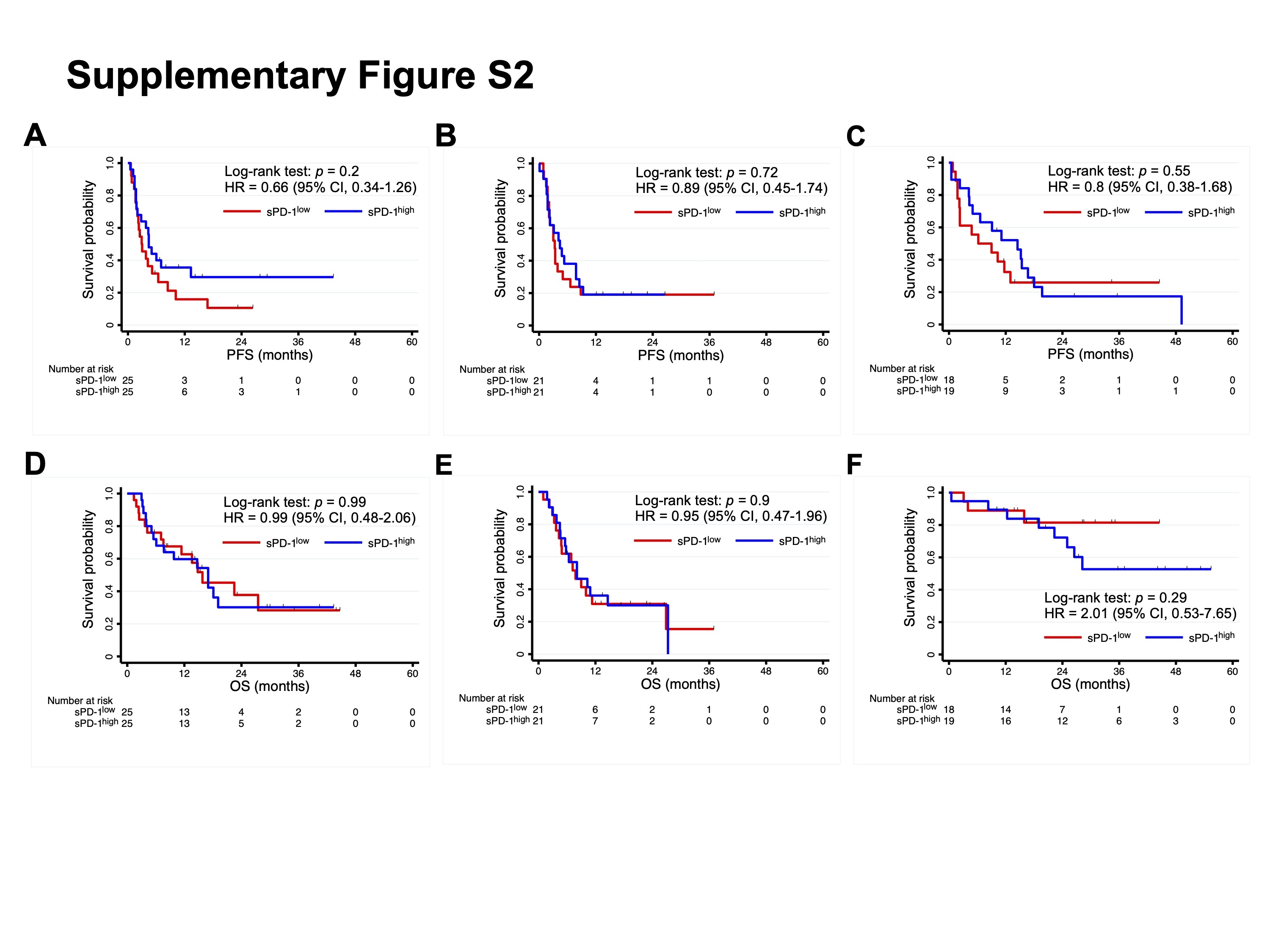

Supplement: Supplementary Figure 2 — Kaplan-Meier curves of PFS (A–C) and OS (D–F) for patients with sPD-1high or sPD-1low levels among individuals with head and neck cancer (A, D), urothelial cancer (B, E), or renal cell cancer (C, F). [file Image_2.jpeg]

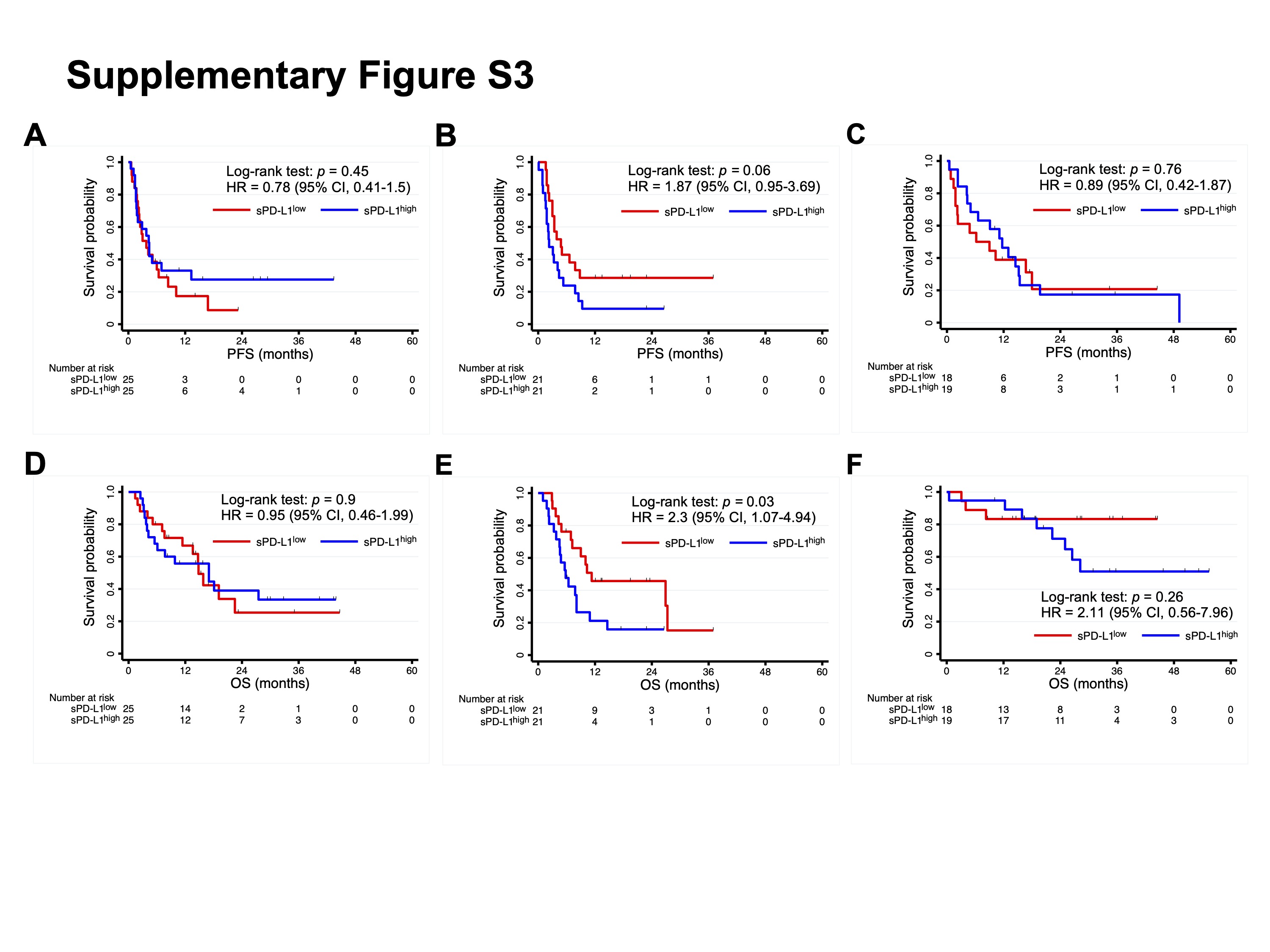

Supplement: Supplementary Figure 3 — Kaplan-Meier curves of PFS (A–C) and OS (D–F) for patients with sPD-L1high or sPD-L1low levels among individuals with head and neck cancer (A, D), urothelial cancer (B, E), or renal cell cancer (C, F). [file Image_3.jpeg]

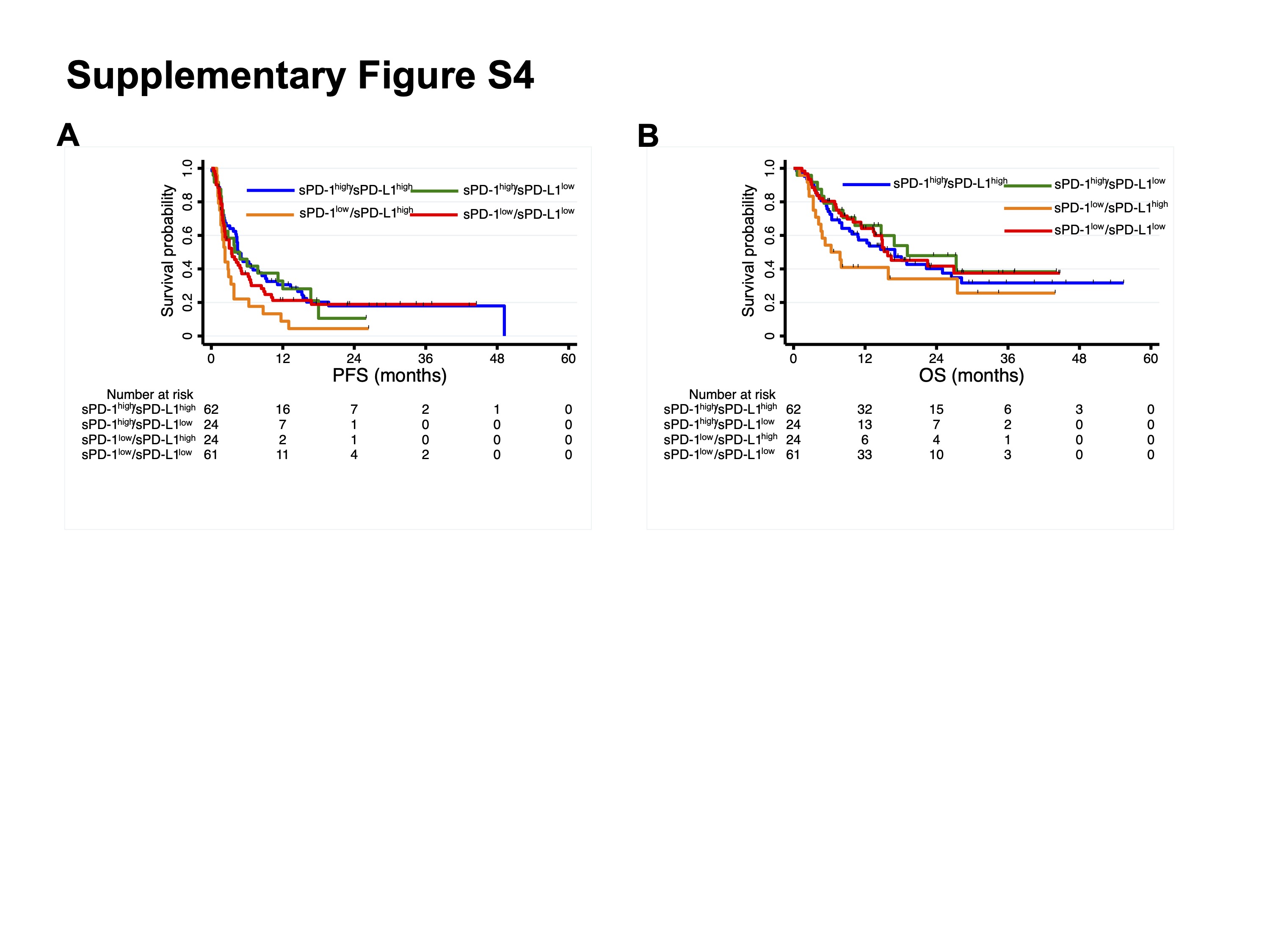

Supplement: Supplementary Figure 4 — Kaplan-Meier curves of PFS (A) and OS (B) for all patients according to combined low or high levels of sPD-1 and sPD-L1. [file Image_4.jpeg]
